# Sequence‐ and Docking‐Site‐Dependent Contributions to Multi‐Site Phosphorylation of an Intrinsically Disordered MAPK Substrate

**DOI:** 10.1002/advs.202503987

**Published:** 2025-06-29

**Authors:** Thibault Orand, Elise Delaforge, Marion Chenal, Maud Tengo, Torsten Herrmann, Juan Cortés, Pau Bernadó, Malene Ringkjøbing Jensen

**Affiliations:** ^1^ Univ. Grenoble Alpes CEA CNRS IBS Grenoble 38044 France; ^2^ LAAS‐CNRS Université de Toulouse CNRS Toulouse 31400 France; ^3^ Centre de Biologie Structurale (CBS) Université de Montpellier INSERM CNRS Montpellier 34090 France

**Keywords:** docking sites, intrinsically disordered protein, local effective concentration, mitogen‐activated protein kinases, NMR, scaffold protein, substrate phosphorylation

## Abstract

Protein kinases often rely on docking site motifs to enhance substrate interactions and facilitate phosphorylation. For example, mitogen‐activated protein kinases (MAPKs) utilize D‐ and F‐motifs, which frequently act in concert to enable bipartite substrate binding. While these motifs are known to modulate phosphorylation efficiency, their quantitative impact on target site phosphorylation within long intrinsically disordered substrates remains largely unexplored. Using NMR spectroscopy, JNK1‐dependent phosphorylation of JIP1, a 450‐amino acid disordered substrate, is investigated, identifying eleven phosphosites with distinct phosphorylation efficiencies. By selectively disrupting JNK1 binding to the D‐ and F‐motifs of JIP1, the determinants of phosphorylation efficiency are uncovered. Specifically, it is found that the D‐motif selectively enhances phosphorylation in the C‐terminal direction in a length‐dependent manner, impressively increasing the phosphorylation efficiencies of sites located at sequence distances exceeding 120 amino acids, while the F‐motif primarily promotes phosphorylation of a site located immediately N‐terminal to the F‐motif. Additionally, docking‐site‐independent phosphorylation is observed, whose efficiency is dictated by the intrinsic sequence preference of JNK1, as inferred from motif scores derived from positional scanning peptide arrays. The work highlights how docking site motifs and sequence context synergistically regulate phosphorylation efficiency, emphasizing the critical role of substrate architecture in determining MAPK‐mediated signaling outcomes.

## Graphical abstract

Real‐time NMR spectroscopy is used to study JNK1‐dependent phosphorylation of the intrinsically disordered substrate JIP1, revealing eleven phosphosites with distinct phosphorylation efficiencies. The site‐specific phosphorylation rates are shown to be determined by both the sequence position of the phosphosites relative to the two kinase docking site motifs within JIP1 as well as the local sequence context surrounding each phosphosite.

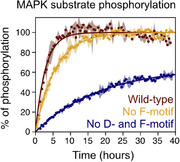

## 1 Introduction

Protein phosphorylation is one of the most common forms of post‐translational modification. It actively regulates many cellular processes such as gene transcription, cell differentiation and proliferation, subcellular trafficking, and apoptosis, mainly by influencing the structure and activity of proteins as well as their interaction networks.^[^ ^1^, ^2^, ^3^, ^4^, ^5^ ^]^ The three‐tiered mitogen‐activated protein kinase (MAPK) cell signaling pathways are conserved in eukaryotes, and they regulate cell responses to a wide range of stimuli including growth factors, oxidative stress, and inflammatory cytokines.^[^ ^6^, ^7^, ^8^, ^9^ ^]^ These stimuli are most often initiated at the level of cell‐membrane receptors and further transduced downstream through a series of phosphorylation events in order to activate specific transcription factors and elicit an adaptive cellular response.

It has become increasingly clear that the regulation of the activity of MAPK signaling modules involves scaffold proteins.^[^ ^10^, ^11^, ^12^, ^13^ ^]^ These proteins function as regulatory hubs by assembling all three components of a MAPK signaling module to optimize their spatial and temporal localization.^[^ ^14^, ^15^, ^16^, ^17^, ^18^ ^]^ The JNK‐interacting protein 1 (JIP1) acts as a scaffold protein in the c‐Jun N‐terminal kinase (JNK) pathway by coordinating the specific recruitment of the three kinases DLK, MKK7, and JNK into a multi‐enzyme complex.^[^ ^19^, ^20^, ^21^, ^22^ ^]^ JIP1 contains a long N‐terminal intrinsically disordered region (IDR), essential for the recruitment of JNK pathway components, that becomes hyperphosphorylated upon JNK stimulation.^[^ ^23^ ^]^ Interestingly, phosphorylation of JIP1 is required for its scaffolding function with JNK phosphorylation of T103 regulating the association of DLK with JIP1.^[^ ^24^ ^]^ In addition to its function as a MAPK scaffold protein, JIP1 is implicated in sub‐cellular trafficking by interacting with kinesin, and phosphorylation of JIP1 at S421 regulates the directionality of microtubular transport in neuronal cells.^[^ ^25^, ^26^, ^27^, ^28^ ^]^ Finally, phosphorylation of T205 has been shown to regulate axonal outgrowth.^[^ ^29^ ^]^ Thus, phosphorylation of JIP1 is crucial for regulating and driving many of its functions.

To achieve specificity in their phosphorylation patterns, MAPKs employ docking strategies to recognize their substrates. This recognition primarily relies on substrate docking site motifs (D‐motifs) that bind to the D‐motif recruitment site (DRS) on the surface of the MAPKs, distant from the catalytic site.^[^ ^30^, ^31^, ^32^, ^33^, ^34^, ^35^, ^36^, ^37^ ^]^ These motifs consist of up to five basic residues followed by three hydrophobic residues, adhering to the consensus sequence: K/R_1‐5_−X_0‐5_−Φ_L_−X_1‐3_−Φ_A_−X−Φ_B_, where X represents any amino acid type and Φ denotes a hydrophobic residue. The three hydrophobic residues are accommodated in three hydrophobic pockets (L, A, and B) on the surface of the MAPK, while the positively charged residues interact with the negatively charged common docking groove. In addition to D‐motifs, MAPKs can recognize F‐motifs, which bind to the F‐motif recruitment site (FRS) located below the catalytic site.^[^ ^38^, ^39^ ^]^ F‐motifs were first identified in the ETS family of transcription factors as phenylalanine‐rich motifs following the consensus sequence FXFP.^[^ ^40^, ^41^, ^42^, ^43^ ^]^ We have previously shown that the IDR of JIP1 contains a JNK1‐specific D‐motif (^159^KRPTTLNLF^167^) as well as a non‐canonical F‐motif (^211^ICLSDELP^218^) leading to predominantly bipartite binding of JIP1, as shown by the crystal structure of the JIP1‐JNK1 complex that we solved at 2.35 Å resolution.^[^ ^44^ ^]^

D‐ and F‐motifs not only offer a molecular mechanism for optimizing the encounter between MAPKs and their substrates, but their sequence position relative to target sites determines the phosphorylation efficiencies. It has been shown that displacing or mutating the D‐ and F‐motifs within the sequence of the Elk‐1 and c‐Jun transcription factors, as well as within the Na^+^/H^+^ exchanger 1, influences the site‐specific MAPK phosphorylation efficiencies.^[^ ^42^, ^45^, ^46^, ^47^ ^]^ However, these studies did not systematically quantify the length‐dependent effects, specifically in terms of determining the sequence distance, both in the N‐terminal and C‐terminal directions, over which different docking site motifs affect the phosphorylation efficiencies of target sites. Elucidating the molecular mechanisms by which MAPK docking sites direct substrate phosphorylation patterns is essential for understanding the precise regulation of cellular signaling pathways and uncovering how their dysregulation contributes to disease.

In this study, we perform real‐time nuclear magnetic resonance (NMR) experiments on the 450‐amino acid IDR of JIP1, in the presence of active JNK1, to provide quantitative, temporal, and site‐specific insights into the phosphorylation of all eleven serine‐proline and threonine‐proline (S/T‐P) sites within JIP1. By introducing point mutations to disrupt JNK1 binding at the D‐ and F‐motifs and using structural ensembles to estimate local effective concentrations of the phosphosites at the kinase active site, we demonstrate that the variability in phosphorylation efficiency across the JIP1 sequence is primarily governed by structural constraints imposed by the D‐motif and to a lesser extent by the F‐motif. The D‐motif selectively promotes phosphorylation in the C‐terminal direction, remarkably enhancing the phosphorylation efficiency of S/T‐P sites located at sequence distances exceeding 120 amino acids. Additionally, we show that docking‐site‐independent phosphorylation of JIP1 is dictated by the intrinsic sequence preference of JNK1. Collectively, our findings highlight how the strategic placement of kinase docking site motifs within substrates can drive efficient phosphorylation of target sites, even in the context of very long intrinsically disordered regions.

## 2 Results

### 2.1 JNK1 Phosphorylates All S/T‐P Sites within the IDR of JIP1

**Figure 1:**
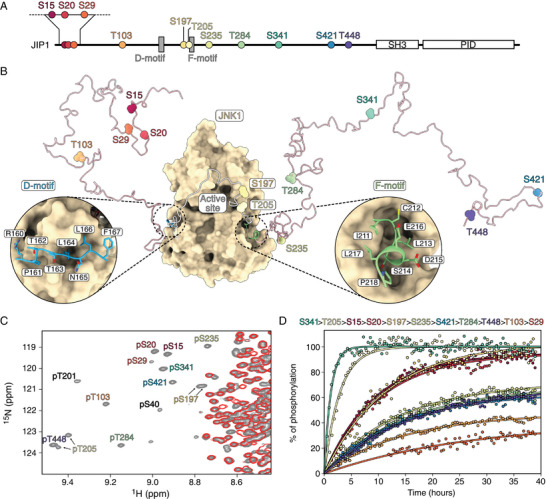
NMR detection of JNK1‐dependent phosphorylation of the IDR of JIP1. A) Domain organization of JIP1 consists of a long N‐terminal IDR, a dimeric Src‐homology 3 (SH3) domain, and a phosphotyrosine interaction domain (PID). The eleven S/T‐P sites in the IDR of JIP1 are indicated, along with the position of the D‐motif (residues 159–167) and F‐motif (residues 211–218). B) Structure of the JIP1‐JNK1 complex (PDB 9FT9), with JNK1 shown in beige and the D‐ and F‐motifs of JIP1 shown in blue and green, respectively. The IDR of JIP1 (residues 1–450) is represented as a single random coil conformation in pink, with the linker between the D‐ and F‐motif in gray. C) Zoom on the ^1^H‐^15^N HSQC spectrum of the IDR of JIP1 before (red) and after (grey) phosphorylation by active JNK1. Labels indicate assignments of the resonances corresponding to phosphorylated residues. Spectra were recorded at 5 °C with a JIP1 concentration of 100 µm. D) Kinetic phosphorylation profiles of the S/T‐P sites in JIP1 obtained from real‐time NMR measurements. Phosphorylation kinetics were derived from NMR resonance intensities in ^1^H‐^15^N HSQC spectra, and normalized to the percentage of phosphorylation at the end of the time series. For sites showing multiple resonances during the time course (S197 and T205), the sum of their intensities is shown. Spectra were recorded at 25 °C with a JIP1 concentration of 100 µm. Phosphorylation kinetics were analyzed using Equation (2) (solid lines), and the sites were ranked by phosphorylation rates, determined as the initial slope (shown in descending order at the top).

JIP1 is an ideal substrate for studying the docking‐site‐dependent contributions as it comprises eleven S/T‐P sites distributed along its 450‐amino acid IDR with a centrally‐located D‐F motif pair (**Figure** **1**A,B). We have previously obtained the NMR spectral assignment of the IDR showing that it adopts an ensemble of random coil conformations with only a modest propensity to form secondary structures.^[^ ^44^ ^]^ The assignment enables us to monitor the phosphorylation events in JIP1 at atomic resolution. Upon the addition of active JNK1, resonances of phosphorylated serines and threonines appear in a distinct region of the ^1^H‐^15^N HSQC spectrum (Figure 1C). These resonances were assigned at 5 °C (Table S1, Supporting Information) using triple resonance experiments of phosphorylated sub‐constructs (Figure S1A, Supporting Information) and by mutagenesis of specific phosphorylation sites in JIP1_1‐266_ (Figure S1B–G, Supporting Information). The assignment of phosphorylated resonances at 25 °C was obtained by comparing HSQC spectra recorded at 5 and 25 °C (Figure S2A, Supporting Information) and further confirmed by overlaying HSQC spectra of various phosphorylated sub‐constructs of JIP1 (Figure S2B, Supporting Information). Our results show that all eleven S/T‐P sites in the IDR are phosphorylated by JNK1. The narrow chemical shift dispersion in the ^1^H dimension is maintained upon phosphorylation showing that the IDR remains disordered upon multi‐site phosphorylation (Figure S3, Supporting Information).

### 2.2 Two Non‐Canonical Sites are Phosphorylated in JIP1

The catalytic site of MAPKs exhibits a strong preference for serine or threonine residues followed by a proline, primarily due to the structural properties of the substrate binding pocket, which forms upon phosphorylation of the MAPK activation loop.^[^ ^48^ ^]^ This pocket serves as an anchor point for the proline residue within the phosphorylation motif.^[^ ^49^, ^50^ ^]^ The preference of the catalytic site for a phosphorylation site is influenced by the surrounding sequence context. While proline is the preferred amino acid, studies have demonstrated that glycine, leucine, phenylalanine, cysteine, and aspartic acids can occupy this position and still support phosphorylation, although with reduced efficiency.^[^ ^51^ ^]^ In addition to the eleven S/T‐P sites phosphorylated by JNK1, we identified two non‐canonical S/T phosphorylation sites (Figure 1C). The first site, S40 (^35^LTHDISLEEF^44^), is followed by a leucine, while the second site, T201 (^196^SSPLKTGEQT^205^) is followed by a glycine. Phosphorylation of S40, but not T201, was previously observed by mass spectrometry analysis of JIP1 from HEK293 cells^[^ ^23^ ^]^ suggesting that at least one of these sites is physiologically relevant.

### 2.3 The S/T‐P Sites in JIP1 are Phosphorylated with Different Efficiencies

To provide a quantitative and temporal characterization of JIP1 phosphorylation, we monitored the phosphorylation state of individual S/T‐P sites in JIP1 using real‐time NMR at 25 °C and pH 7.1. Active JNK1 was added to an NMR sample of ^15^N‐labeled JIP1, and the intensities of the peaks corresponding to the phosphorylated residues were measured over a 40‐h period through a series of ^1^H‐^15^N HSQC spectra (Figure S4, Supporting Information). Quantifying the peak intensities was not straightforward as the chemical shifts of the resonances corresponding to phosphorylated residues changed significantly over time with threonines displaying larger chemical shift perturbations than serines (Figure S5A,B, Supporting Information). These chemical shift changes were attributed to a gradual decrease in pH in the NMR sample over time, resulting from ATP hydrolysis occurring both with and without phosphoryl transfer (Figure S5C,D, Supporting Information).^[^ ^52^ ^]^ This small pH change (0.1 pH unit) primarily affected the chemical shifts of the amide groups of the phosphorylated residues, as even minor pH variations can significantly alter the population of amide protons involved in hydrogen bonding with the side chain phosphate groups.^[^ ^53^ ^]^

To analyze our data, consisting of 90–95 individual ^1^H‐^15^N HSQC spectra, we employed in‐house written software to automatically trace the peaks and quantify resonance intensities in each spectrum. To determine the degree of phosphorylation of each S/T‐P site at the end of the time series, we measured the intensity ratio of the resonances corresponding to the non‐phosphorylated residues in ^1^H‐^15^N HSQC spectra acquired at 5 °C before and after the phosphorylation reaction (either of the S/T residue itself or the preceding residue, see Experimental Section). This approach allows to extract the kinetic profile of each phosphorylation site in a quantitative manner.

The profiles of each S/T‐P site show markedly different phosphorylation efficiencies (Figure 1D). For example, S341 and T205 are phosphorylated rapidly, while S29 and T103 show significantly slower phosphorylation rates. The phosphorylation kinetics of all eleven S/T‐P sites show no discernible latency phase. Analysis of the data using Equation (2) (see Experimental Section) shows that some sites fail to reach full phosphorylation, likely due to ATP depletion resulting from intrinsic ATP hydrolysis by JNK1. Moreover, the phosphorylation pattern in the JIP1 sub‐constructs, JIP1_116‐266_, JIP1_1‐266_ and JIP1_1‐372_, are qualitatively the same as in JIP1_1‐450_ (Figure S1A, Supporting Information), and alanine substitutions of residues S15, S29, T103, S197, T201 and T205 do not prevent phosphorylation at other sites (Figure S1B–G, Supporting Information). Taken together, these results demonstrate the absence of strong cooperativity between the phosphorylation sites in JIP1.

### 2.4 JNK1 Employs a Distributive Mechanism to Phosphorylate JIP1

The advantage of NMR spectroscopy for characterizing protein phosphorylation is that clusters of phosphorylated residues can be entirely resolved and their interdependence studied in detail.^[^ ^54^, ^55^, ^56^, ^57^, ^58^ ^]^ In our experiments, S197, T201, and T205 undergo phosphorylation with each residue exhibiting distinct chemical shift signatures depending on the phosphorylation state of their neighbors. T205, S197, and T201 show three, two, and one NMR resonance(s), respectively, over the course of the phosphorylation reaction (Figure S4, Supporting Information) showing that JNK1 phosphorylates this cluster in a defined order T205 → S197 → T201. By analyzing the time dependence of the intensities of the three resonances of T205, we determined the apparent rate constants for each phosphorylation event within the cluster (Figure S6, Supporting Information). The much lower concentration of JNK1 relative to JIP1, combined with the appearance of intermediate species in the NMR spectra over time, suggest that JNK1 dissociates from JIP1 after each phosphorylation event, consistent with a distributive phosphorylation mechanism.^[^ ^59^ ^]^

Full‐length JIP1 is a dimer, with dimerization being mediated by its SH3 domain.^[^ ^60^, ^61^, ^62^ ^]^ To study the impact of dimerization on the phosphorylation kinetics of the S/T‐P sites within the IDR, we engineered a construct of JIP1 containing the complete IDR (residues 1–450) linked to the SH3 domain of JIP1 (residues 489–553) via a (GS)_3_G linker (hereafter named JIP1^SH3^, Figure S7A, Supporting Information). We opted for a glycine‐serine‐rich linker instead of the native JIP1 sequence, as residues 451‐488 of JIP1 are prone to aggregation. Size exclusion chromatography coupled to detection by multi‐angle laser light scattering (SEC‐MALLS) confirms that JIP1^SH3^ is a dimer in solution (Figure S7B, Supporting Information). Moreover, the ^1^H‐^15^N HSQC spectra of JIP1 and JIP1^SH3^ are very similar suggesting that dimerization does not alter the conformational ensemble of the IDR (Figure S7C, Supporting Information). Phosphorylation of JIP1^SH3^ by active JNK1 shows that the target sites are the same as in JIP1 (Figure S7D, Supporting Information) and that the phosphorylation rates are unchanged in the dimer, except for T448 which is located close to the SH3 domain in the sequence (Figure S7E, Supporting Information). Taken together, our data show no evidence for cross‐monomer phosphorylation providing further support for a distributive phosphorylation mechanism of the JIP1 IDR.

### 2.5 The F‐Motif Mainly Impacts Phosphorylation of T205

**Figure 2:**
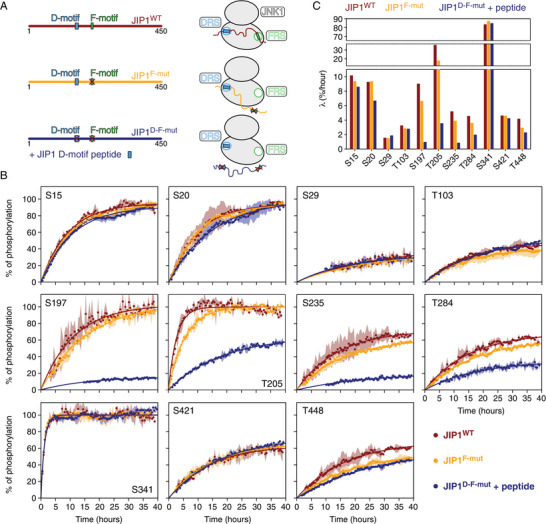
The D‐ and F‐motifs direct the phosphorylation pattern of JIP1. A) A schematic overview of the constructs employed in the phosphorylation assays and their binding modes with the JNK1 kinase. B) Kinetic phosphorylation profiles for each of the eleven S/T‐P sites in JIP1 extracted from NMR resonance intensities normalized to the percentage of phosphorylation at the end of the time series. Profiles are shown for wild‐type JIP1 (JIP1^WT^, red), a mutant of JIP1 where JNK1 binding at the F‐motif is disrupted (JIP1^F‐mut^, orange) and a mutant of JIP1 where JNK1 binding to both the F‐ and D‐motifs is abolished, measured in the presence of a peptide corresponding to the D‐motif (JIP1^D‐F‐mut^ + peptide, blue). Each profile represents the average of two biological replicates, with the shadings (light red, light orange, and light blue) indicating standard deviations between measurements. The ^1^H‐^15^N HSQC spectra were recorded at 25 °C with a JIP1 concentration of 100 µm. Phosphorylation kinetics for each S/T‐P site were analyzed using Equation (2) (solid lines). C) Phosphorylation rates (λ, determined as initial slopes of the kinetic phosphorylation profiles) of the eleven phosphorylation sites in JIP1. The rates are reported for three different experimental conditions: JIP1^WT^ (red), JIP1^F‐mut^ (orange), and JIP1^D‐F‐mut^ + peptide (blue).

Several factors may influence the apparent phosphorylation rate of each of the eleven S/T‐P sites in JIP1 (Figure 1D). These include the position of the phosphosite with respect to the D‐ and F‐motifs,^[^ ^42^, ^45^, ^46^, ^47^ ^]^ the local primary sequence surrounding the S/T‐P site^[^ ^47^, ^48^, ^51^, ^63^ ^]^ and allosteric modulation of the kinase activity through D‐motif binding to the DRS.^[^ ^32^, ^64^, ^65^ ^]^ To identify the key factors governing the observed phosphorylation rates, we aimed to selectively disrupt JNK1 binding to the D‐ and F‐motifs, either individually or in combination, and assess the resulting impact on the phosphorylation efficiencies across all eleven S/T‐P sites in JIP1 (**Figure** **2**A).

The F‐motif in JIP1 exhibits a low intrinsic binding affinity for JNK1 (*K*_d_ = 2.2 mm).^[^ ^44^ ^]^ However, the presence of the high‐affinity D‐motif (*K*_d_ = 81 nm) significantly enhances the local effective concentration of the F‐motif at the FRS, resulting in a high occupancy (78%) of the FRS.^[^ ^44^ ^]^ Previously, we demonstrated that JNK1 binding to the F‐motif can be abolished through a double mutation in JIP1 (I211A/L213A, hereafter referred to as JIP1^F‐mut^, Figure 2A), while maintaining the ability of JNK1 to bind the D‐motif.^[^ ^44^ ^]^ Comparing the kinetic phosphorylation profiles of all eleven S/T‐P sites in wild‐type JIP1 (JIP1^WT^) to those of JIP1^F‐mut^ reveals a relatively weak impact of the F‐motif on shaping the phosphorylation landscape of JIP1 (Figure 2B, red and yellow profiles). Among all sites, T205 shows the most pronounced reduction in phosphorylation rate, decreasing by 2‐fold (Figure 2C; Table S2, Supporting Information). Smaller reductions of 1.2‐ to 1.4‐fold are observed for the phosphorylation rates of S197, S235, T284, and T448, while the remaining sites show nearly identical rates (Figure 2C). These findings align with previous studies showing that F‐motifs preferentially enhance the phosphorylation efficiency of target sites located 10–15 amino acids upstream of the F‐motif.^[^ ^40^, ^41^, ^42^ ^]^ Our results indicate that the F‐motif in JIP1 imposes favorable structural constraints, facilitating productive encounters of T205 with the kinase active site. However, we note that T205 phosphorylation remains highly efficient even in the absence of the F‐motif. This observation suggests that the high‐affinity D‐motif alone may be sufficient to drive optimal phosphorylation of target sites, including T205, and that regulation of substrate phosphorylation may not be the sole function of the F‐motif in JIP1.

### 2.6 The D‐Motif Strongly Directs the Phosphorylation Pattern of JIP1

**Figure 3:**
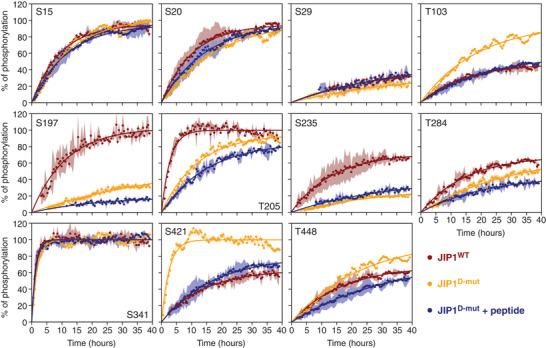
Kinetic phosphorylation profiles for each of the eleven S/T‐P sites in JIP1 derived from NMR resonance intensities normalized to the percentage of phosphorylation at the end of the time series. Phosphorylation kinetics are shown for wild‐type JIP1 (JIP1^WT^, red, average of two biological replicates, standard deviation in light red), a mutant of JIP1 where JNK1 binding to the D‐motif was disrupted (JIP1^D‐mut^, orange, single experiment, uncertainty estimated as twice the noise level in each plane, light orange shading) and the same mutant in the presence of a peptide corresponding to the D‐motif of JIP1 (JIP1^D‐mut^ + peptide, blue, average of two biological replicates, standard deviation in light blue). The ^1^H‐^15^N HSQC spectra were recorded at 25 °C with a JIP1 concentration of 100 µm, and the intensities were analyzed for each S/T‐P motif using Equation (2) (solid lines).

To investigate the effect of the D‐motif on the phosphorylation rates of the S/T‐P sites in JIP1, we introduced mutations in key residues of the D‐motif (R160E, P161A, L164A, and L166A, hereafter referred to as JIP^D‐mut^, Figure 2A). These mutations effectively disrupt JNK1 binding to the D‐motif while preserving weak binding affinity (*K*_d_ = 2.2 mm) to the F‐motif.^[^ ^44^ ^]^ We compared the kinetic phosphorylation profiles of JIP1^WT^ and JIP^D‐mut^ revealing decreases in the phosphorylation efficiencies of S197, T205, S235, and T284 (**Figure** **3**). Unexpectedly, however, significant increases in the phosphorylation rates of T103, S421, and T448 were also observed. These findings suggest that the unoccupied DRS can transiently interact with specific hydrophobic clusters along the JIP1 sequence, enhancing the phosphorylation efficiencies of phosphosites favorably positioned relative to these clusters. This hypothesis is supported by NMR resonance intensities of JIP1^D‐mut^ in the presence of inactive JNK1 (Figure S8, Supporting Information). To circumvent this redistribution of kinase‐substrate interactions, we measured the kinetic profiles of JIP^D‐mut^ in the presence of a D‐motif peptide (^157^RPKRPTTLNLF^167^), which binds to JNK1 with high affinity (*K*_d_ = 217 nm).^[^ ^44^ ^]^ This approach not only ensures full occupancy of the DRS pocket during the kinetic experiments thereby minimizing redistribution of kinase‐substrate interactions (Figure S8, Supporting Information), but also accounts for any potential allosteric contributions to the catalytic activity of JNK1 from D‐motif binding. As shown in Figure 3, the D‐motif peptide effectively eliminates the elevated phosphorylation rates observed when comparing JIP1^D‐mut^ to JIP1^WT^. Consequently, this approach was adopted consistently when mutating the D‐motif of JIP1.

To assess the impact of the D‐motif on the phosphorylation efficiency of target sites in JIP1, we compared the kinetic phosphorylation profiles of JIP1^F‐mut^ with those of a JIP1 variant where both the D‐ and F‐motif were mutated (JIP1^D‐F‐mut^), again adding the D‐motif peptide to prevent redistribution of kinase‐substrate interactions (Figure 2A). The profiles reveal two key insights. First, the phosphorylation rates of S197, T205, S235, and T284, located immediately downstream of the D‐motif, are significantly slower in JIP1^D‐F‐mut^ + peptide than in JIP1^F‐mut^ (Figure 2B, yellow and blue profiles). This identifies proximity‐induced phosphorylation^[^ ^66^ ^]^ as a major contribution to the phosphorylation efficiencies of these residues in JIP1^WT^. Notably, the influence of proximity‐induced phosphorylation attenuates with increasing distance from the D‐motif, as evidenced by similar phosphorylation rates of S341 and S421 in JIP1^F‐mut^ and JIP1^D‐F‐mut^ + peptide. Second, the phosphorylation rates of S15, S20, S29, and T103 are nearly identical in JIP1^F‐mut^ and JIP1^D‐F‐mut^ + peptides (Figure 2B). This observation aligns with the preferential orientation of bound kinases by D‐motifs, which position residues C‐terminal to the D‐motif closer to the active site than those located N‐terminally.^[^ ^42^, ^47^ ^]^ For example, while T103 and T205 are both located ≈50 residues away from the D‐motif, T205 shows a markedly greater reduction in phosphorylation rate in JIP1^D‐F‐mut^ + peptide compared to JIP1^F‐mut^ (Figure 2B,C).

Collectively, our results demonstrate that the D‐motif plays a critical role in directing the specific phosphorylation pattern of JIP1, with proximity‐induced phosphorylation being a dominant contribution to the observed phosphorylation rates of sites located C‐terminal to the D‐motif. The efficiency of this mechanism decreases with increasing sequence distance from the D‐motif, essentially becoming negligible for residues located more than 120 amino acids from the D‐motif. Finally, the D‐motif imposes a strong directionality, as phosphorylation rates of sites on the N‐terminal side of the D‐motif exhibit little to no dependence on its presence.

### 2.7 The Influence of the D‐Motif on Phosphorylation Rates can be Predicted by Local Effective Concentrations

**Figure 4:**
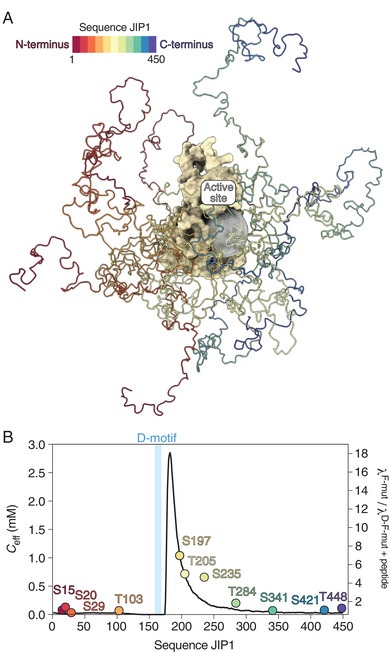
The impact of the D‐motif on the phosphorylation rates correlates with local effective concentrations (*C*_eff_) of phosphosites at the active site of JNK1. A) Structural ensemble of the JIP1‐JNK1 complex, with JIP1 anchored to JNK1 via the D‐motif. The JNK1 active site is depicted as a gray sphere with a radius of 15 Å corresponding to the radius used for the *C*_eff_ calculations. Five representative conformations of JIP1 are shown. B) Calculated *C*_eff_ values for all residues in JIP1 derived from the structural ensemble of the JIP1‐JNK1 complex with JIP1 anchored to JNK1 via the D‐motif (black line, left *y*‐axis). The *C*_eff_ values are compared to the ratio of the phosphorylation rates (λ, defined as the initial slope of the kinetic phosphorylation profiles) between JIP1^F‐mut^ and JIP1^D‐F‐mut^ + peptide for all eleven S/T‐P sites in JIP1 (circles, right *y*‐axis).

Tethering of kinases to their substrates has been shown to significantly enhance the rate of substrate phosphorylation in a way that depends on the local effective concentration of the phosphosite at the kinase active site, dictated by the specific linker sequence.^[^ ^67^, ^68^, ^69^, ^70^ ^]^ Building on this concept, we sought to quantify how the D‐motif enhances the local effective concentration of the S/T‐P sites in JIP1 at the active site of JNK1. To achieve this, we generated structural ensembles of the JIP1‐JNK1 complex, anchoring JIP1 at its D‐motif while allowing the remaining chain to adopt random coil conformations that avoided steric clashes with JNK1 (**Figure** **4**A). A total of 400,000 conformations were generated, and the local effective concentration, *C*_eff_, of each residue in JIP1 at the active site of JNK1 was calculated (Equation (3), see Experimental Section). Consistent with our experimental data, the D‐motif selectively enhances the local effective concentrations of residues located C‐terminal to the D‐motif. The highest value of *C*_eff_, 2.8 mm, is observed at residue 182, followed by a gradual decay extending to approximately residue 300, beyond which *C*_eff_ approaches 0 mm (Figure 4B). This trend aligns with the experimentally observed influence of the D‐motif on the phosphorylation rates, as shown by a qualitative comparison of the calculated *C*_eff_ values and the ratios of the phosphorylation rates between JIP1^F‐mut^ and JIP1^D‐F‐mut^ + peptide (Figure 4B). Overall, our calculations demonstrate that the structural constraints imposed by D‐motif binding shape the structural ensemble of JIP1, governing the efficacy of proximity‐induced phosphorylation across an unexpectedly large sequence range.

We also calculated the local effective concentration in JIP1 when both the D‐ and F‐motifs are simultaneously tethered to JNK1 (Figure S9, Supporting Information). As expected, residues located in the linker between the D‐ and F‐motifs exhibit very high effective concentrations, consistent with their frequent proximity to the JNK1 active site. However, this finding contrasts with the modest impact of the F‐motif on phosphorylation efficiency, suggesting that, in this case, a high local concentration alone is not sufficient to ensure efficient phosphoryl transfer. One possible explanation is that the phosphorylation sites within the linker (S197, T205) are hindered from adopting productive orientations at the kinase active site for catalysis due to structural constraints imposed on the linker region by the bipartite binding of JIP1. Alternatively, phosphorylation efficiency may have reached a plateau level, beyond which further increases in the local effective concentration no longer result in a significant gain. The D‐motif may already elevate the effective substrate concentration to near this plateau so that the additional contribution from the F‐motif only yields marginal improvements in the phosphorylation of the linker sites.^[^ ^69^ ^]^

### 2.8 Docking‐Site‐Independent Phosphorylation Depends on JNK1 Motif Preferences

By simultaneously disrupting binding to both the D‐ and F‐motifs, we can study the contribution of docking‐site‐independent interactions to the phosphorylation rates of each S/T‐P site in JIP1. The determined phosphorylation rates vary considerably across different sites, notably with S15, S20, and S341 showing significantly faster rates than any other site within the JIP1 sequence (Figure 2B, blue profiles). This prompted us to determine whether this rate variation can be attributed to the intrinsic sequence preferences of JNK1 for the various phosphosites.

**Figure 5:**
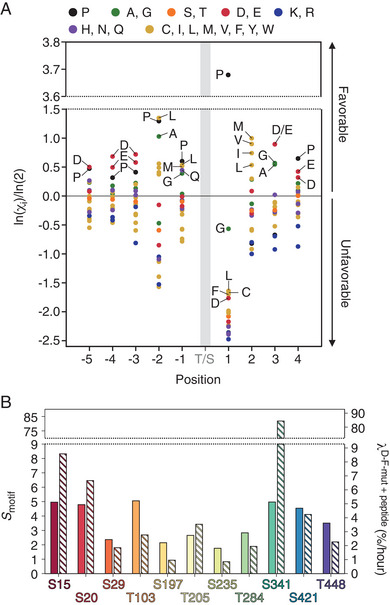
The docking‐site‐independent phosphorylation rates correlate with the intrinsic sequence preference of JNK1. A) Bit scores of the selectivity value, χ_i_, for the twenty amino acids at each position (*i*) surrounding the phosphosite (position 0). These data were previously obtained from positional scanning peptide arrays profiling the substrate specificities of JNK1.^[^ ^51^ ^]^ B) Motif scores, *S*_motif_, calculated for the eleven S/T‐P sites in JIP1 (left *y*‐axis, filled bars) compared to the phosphorylation rates (λ, defined as the initial slope of the kinetic phosphorylation profiles) of JIP1^D‐F‐mut^ + peptide (right *y*‐axis, hatched bars).

Recently, positional scanning peptide arrays were used to profile the substrate specificities of the human serine/threonine kinome, including JNK1.^[^ ^51^ ^]^ The study systematically substituted each of the 20 amino acids at nine positions surrounding a central phospho‐acceptor site (serine or threonine), assessing their effect on enzymatic activity. This approach allowed us to assign a selectivity value, χ*_i_*, to each amino acid at every position (*i*) within the phosphorylation motif, as explained previously.^[^ ^51^ ^]^ The selective values for JNK1, available in reference^[^ ^51^ ^]^ and shown in **Figure** **5**A, reveal a very strong preference for proline at position 1, but also highlight a preference for hydrophobic residues, primarily aliphatic ones, at position 2, and negatively charged residues or small side‐chain amino acids (such as alanine or glycine) at position 3. Finally, prolines and leucines are preferred at positions −1 and −2, while negatively charged residues or prolines are favored at positions −3, −4, and −5.

A motif score, *S*_motif_, is calculated by summing the bit scores for each position surrounding the phospho‐acceptor site (as implemented at https://kinase‐library.phosphosite.org/site)^[^ ^71^ ^]^:

(1) $$S_{\mathrm{motif}}=\sum_{i=-5}^{i=4}\ln\left(\chi_{i}\right)/\ln\;\left(2\right)$$

Since JNK1 shows almost no sequence preference between serine and threonine at position 0, we set χ_0_ to 1.^[^ ^51^ ^]^ We compared the calculated motif scores for the eleven S/T‐P sites in JIP1 with the measured phosphorylation rates in JIP1^D‐F‐mut^ + peptide (Figure 5B). The results reveal a correlation between the phosphorylation rates and the intrinsic sequence preference of JNK1. Specifically, sites with low to medium motif scores (*S*_motif_ < 4) show a strong correlation with phosphorylation rates, however, this agreement weakens for sites predicted to be highly favorable JNK1 substrates (*S*_motif_ > 4) (Figure S10A, Supporting Information). Notably, S341 stands out as an outlier due to its exceptionally rapid phosphorylation rate (Figure 5B). These observations suggest that motif score calculations effectively predict phosphorylation rates for low to moderately favorable sites in JIP1. While highly favorable sites can be identified using this score, it is less reliable for directly predicting the phosphorylation rates. Supporting this conclusion, we observe an excellent agreement between the motif score and the percentage of phosphorylation (Figure S10B,C, Supporting Information). Comparing the percentage of phosphorylation, rather than the rates directly, essentially equalizes all sites with high motif scores.

The use of the motif score is essential for explaining differences in phosphorylation efficiencies among sites with similar neighboring amino acids. For example, the phosphorylation rate of S29 is significantly slower than that of S15 and S20, despite the immediate neighbors of the phospho‐acceptor sites being highly similar (^13^AASPP^17^, ^18^AASPF^22^, and ^27^IASPP^31^). Motif score calculations reveal that the inefficiency of S29 phosphorylation arises from the presence of unfavorable residues at specific positions: leucine at −4, histidine at −3, asparagine at +3, and phenylalanine at +4. These residues contribute negative bit scores, highlighting their inhibitory impact on the phosphorylation efficiency of S29.

In summary, docking‐site‐independent phosphorylation is observed for all eleven S/T‐P sites and the observed variation in phosphorylation rates across the JIP1 sequence can be attributed to the intrinsic sequence preferences of JNK1.

## 3 Discussion

NMR spectroscopy is a preferred method for site‐specific characterization of protein post‐translational modifications, such as phosphorylation.^[^ ^54^, ^55^, ^72^, ^73^, ^74^, ^75^, ^76^ ^]^ NMR enables precise identification of phosphorylated serine and threonine residues, even in hyper‐phosphorylated proteins. In this study, we employed NMR to obtain a comprehensive characterization of JNK1‐dependent multi‐site phosphorylation of the IDR of JIP1 revealing eleven canonical (S/T‐P) and two non‐canonical phosphorylation sites. A previous study utilizing 2D electrophoresis and mass spectrometry also investigated the phosphorylation landscape of JIP1, however, it lacked temporal resolution and encountered difficulties in unambiguously identifying closely spaced phosphorylation sites within the sequence.^[^ ^24^ ^]^ These limitations were entirely overcome in the present work thereby providing kinetic phosphorylation profiles of all eleven S/T‐P sites in JIP1.

The phosphorylation efficiency can vary significantly across different phosphosites within a substrate. This variation may arise from the degree of complementarity between the flanking sequence of the phosphosite and the structural and chemical properties of the catalytic site of the enzyme, as well as kinase docking interactions that modulate the effective concentration of the phosphosite near the catalytic site. The 450‐amino acid disordered tail of JIP1 studied in this work provides an unprecedented view of how sequence and docking‐site‐dependent factors influence phosphorylation kinetics. This is enabled by the particular architecture of JIP1, featuring a centrally located D‐F‐motif pair and multiple phosphorylation sites distributed along the sequence on both the N‐ and C‐terminal sides of the docking sites. We find that the D‐motif of JIP1 plays a pivotal role in shaping its phosphorylation landscape, exerting a length‐dependent influence on the phosphorylation kinetics of S/T‐P sites located up to an impressive 120 amino acids in the C‐terminal direction from the D‐motif (Figure 2). This long‐range effect is particularly striking given the highly dynamic nature of JIP1 and its extensive local conformational sampling due to the absence of secondary structures.^[^ ^44^ ^]^ To further support the existence of this long‐range effect, we generated structural ensembles of the JIP1‐JNK1 complex, anchoring JIP1 to JNK1 via the D‐motif. Calculations of local effective concentrations at the active site of JNK1 from these ensembles reveal that the D‐motif enhances the effective concentration of residues up to around 140 amino acids in the C‐terminal direction, in a length‐dependent manner. This observation aligns closely with the experimentally determined impact of the D‐motif on the site‐specific phosphorylation rates (Figure 4B).

A previous NMR study investigating ERK2‐dependent phosphorylation of the transcription factor Elk‐1 identified eight phosphosites within the C‐terminal transcriptional activation domain. These sites are located C‐terminal to the D‐motif, with six positioned N‐terminal to the F‐motif.^[^ ^45^ ^]^ Deletion of the D‐motif resulted in reduced phosphorylation rates for sites located up to 50 residues away from the deleted D‐motif, no change in the phosphorylation rate for the site located 65 residues away, and increased phosphorylation rates for residues located between 70 and 105 residues away. The variations in phosphorylation rates suggest that the influence of the D‐motif in promoting Elk‐1 phosphorylation is short‐range compared to the longer‐range effects observed in the present study for JIP1. Interestingly, some phosphosites in Elk‐1 exhibit increased phosphorylation rates upon D‐motif deletion, a phenomenon also observed in JIP1 upon D‐motif mutation when the phosphorylation kinetics are measured without the presence of the D‐motif peptide to saturate the hydrophobic DRS (Figure 3).

Another interesting finding is the strong directionality of the observed proximity‐induced phosphorylation. Only S/T‐P sites located C‐terminal to the D‐motif display proximity‐induced phosphorylation, whereas the phosphorylation rates of sites located N‐terminal to the D‐motif remain unaffected by its presence. This directionality is consistent with structural constraints of the kinase, considering the N‐ to C‐terminal binding orientation of the D‐motif at the DRS and the spatial positioning of the DRS relative to the active site. These observations align with our calculations of local effective concentrations, which reveal that only C‐terminal sites experience enhanced effective concentrations. Importantly, our use of structural ensembles for these calculations provides a significant advantage over polymer physics models,^[^ ^77^ ^]^ which would fail to capture this asymmetry.

The strong directionality imposed by the D‐motif was also observed in the JNK1‐dependent phosphorylation of the transcription factor c‐Jun.^[^ ^47^ ^]^ In c‐Jun, four phosphorylation sites (S63, S73, T91, and T93) are located C‐terminal to the D‐motif (residues 32–50). Transposing the D‐motif to positions either between the phosphorylation sites or C‐terminal to them revealed a strong correlation between the location of the D‐motif and the site‐specific phosphorylation efficiencies. Specifically, the phosphorylation rates were significantly enhanced only for sites positioned C‐terminal to the D‐motif. This directional dependency is further supported by studies on ERK1 phosphorylation of peptide substrates. By designing different peptides, and attaching D‐motifs to a substrate sequence via a linker, it was shown that efficient phosphorylation occurred exclusively in peptides with an N‐terminal D‐motif.^[^ ^78^ ^]^

We also investigated the role of the F‐motif in directing the JIP1 phosphorylation pattern. Our results demonstrate that the F‐motif enhances the phosphorylation rates of nearby sites, particularly T205, located immediately N‐terminal to the F‐motif (Figure 2). The position of the FRS below the active site of the kinase has been shown to favor phosphorylation of S/T‐P sites located N‐terminal to the F‐motif.^[^ ^79^ ^]^ For example, the ETS transcription factor Lin‐1 contains an F‐motif positioned C‐terminal to multiple ERK2 phosphorylation sites.^[^ ^40^ ^]^ Mutations in the F‐motif, and especially its deletion, significantly impair the ERK2‐dependent phosphorylation of Lin‐1. Another example is the cyclic nucleotide phosphodiesterase PDE4D3, which contains both a D‐ and an F‐motif flanking the target serine residue, S579. Mutations in these motifs demonstrate that both docking motifs are required for efficient phosphorylation of PDE4D3 at S579.^[^ ^80^ ^]^ Similarly, the transcription factor Ets‐1 also relies on a bipartite binding mode, however, exploiting two non‐canonical docking sites engaging with the DRS and FRS of ERK2. The docking sites flank the phosphosite T38, which is essential for driving the transactivation of Ets‐1. The bipartite binding mode optimally positions T38 at the active site of ERK2, ensuring its efficient phosphorylation.^[^ ^81^ ^]^ Finally, substrates can exhibit even more complex D‐ and F‐motif compositions, as illustrated by the Na+/H+ exchanger 1 for which NMR studies identified a D‐motif and two F‐motifs within its intrinsically disordered C‐terminus. In this case, the phosphorylation rates of target sites depend on the intricate interplay between the number and positioning of these docking motifs.^[^ ^46^ ^]^

In addition to examining the contributions of docking site‐dependent mechanisms to phosphorylation, we also investigated the role of the local sequence context in determining the phosphorylation efficiency of target sites. Our findings reveal significant docking‐site‐independent phosphorylation of JIP1, with rates correlating with the intrinsic sequence preferences of JNK1 (Figure 5; Figure S10, Supporting Information). Notably, while the phosphorylation of several sites, including S197, T205, S235, and T284, is regulated by the two docking site motifs, other sites appear to be phosphorylated independently, relying primarily on the local sequence context. Interestingly, this includes T103, whose phosphorylation has been shown to be essential for activating the JNK pathway.^[^ ^24^ ^]^

## 4 Conclusion

In conclusion, we have provided a detailed description of the phosphorylation landscape of JIP1 by dissecting the relative contributions of docking‐site‐dependent and independent mechanisms to the phosphorylation of multiple sites throughout the IDR of JIP1. Our work demonstrates how docking site motifs and sequence context synergistically regulate phosphorylation efficiency, emphasizing the critical role of substrate architecture in determining MAPK‐mediated signaling outcomes. More broadly, our results highlight the value of structural ensemble descriptions for predicting the impact of docking site motifs on target site phosphorylation efficiency, as well as the use of motif score calculations to assess docking site‐independent contributions. These insights have implications for synthetic biology, where scaffold protein engineering is of considerable interest.^[^ ^82^, ^83^, ^84^, ^85^, ^86^ ^]^ By identifying key determinants of scaffold protein phosphorylation, our study may inform the rational design of more effective scaffolds for efficient rewiring of cellular signaling networks.

## 5 Experimental Section

### Expression and Purification of JIP1 Constructs and Inactive JNK1

Expression and purification of the various constructs of the JIP1 IDR and inactive JNK1 were carried out as described previously.^[^ ^44^ ^]^ The JIP1^SH3^ construct connects the IDR region of JIP1 (residues 1–450) to its SH3 domain (residues 489–553) via a (GS)_3_G linker. This construct was cloned into a pET‐28a vector with an N‐terminal thioredoxin tag and a 6xHis tag, followed by a tobacco etch virus (TEV) protease cleavage site. Expression and purification of this construct followed the same protocol as used for other JIP1 IDR constructs. Following TEV cleavage, all proteins contained a GHM extension at the N‐terminus.

### SEC‐MALLS Experiments

Size‐exclusion chromatography coupled with multi‐angle laser light scattering (SEC‐MALLS) experiments were conducted at 4 °C on an HPLC consisting of a degasser DGU‐20AD, a LC‐20AD pump, an autosampler SIL20‐ACHT, a communication interface CBM‐20A and a UV–vis detector SPD‐M20A (Schimadzu, Kyoto, Japan), a column oven XL‐Therm (WynSep, Sainte Foy d'Aigrefeuille, France), a static light scattering miniDawn Treos, and a refractive index Optilab rEX detector. 20 µL of JIP1^SH3^ at 5.6 mg mL^−1^ were injected at 0.5 mL min^−1^ on a Superdex 200 10/300 GL (GE Healthcare) equilibrated with 50 mm HEPES pH 7.1, 150 mm NaCl, 2 mm DTT. Data analysis was carried out with the software ASTRA, v5.4.3.20 (Wyatt, Santa‐Barbara, USA).

### In Vitro Phosphorylation Assay

Phosphorylation reactions of JIP1 constructs were performed with ^15^N‐ or ^15^N/^13^C‐isotopically labeled proteins at a concentration of 100 µm (or 50 µm for the JIP1^SH3^ dimer to maintain an equivalent number of JIP1 IDRs as in the monomeric state). Reactions were carried out at 25 °C in the following NMR buffer: 50 mm HEPES pH 7.1, 150 mm NaCl, 2 mm dithiothreitol (DTT), 10 mm MgCl_2_, 5% D_2_O, and with 5 mm adenosine 5’‐triphosphate (ATP). Each reaction consisted of 150 µL of sample (3 mm NMR tubes), to which 5 µL of 45 kDa recombinant, double‐phosphorylated JNK1α1 was added. The kinase stock solution (0.75 mg mL^−1^ with an activity of 89 U mg^−1^ as determined with ATF2 as a substrate at 30 °C) was in a buffer composed of 50 mm Tris/HCl pH 7.5, 150 mm NaCl, 0.1 mm ethylenediaminetetraacetic acid (EDTA), 0.03% Brij‐35, 270 mm sucrose, 1 mm benzamidine, 0.2 mm PMSF, 0.1% 2‐mercaptoethanol (Merck Millipore, Item #14‐327M). The amount of kinase was optimized to obtain measurable phosphorylation rates while ensuring the reaction proceeded slowly enough to capture the initial steps accurately.

The peptide corresponding to the D‐motif of JIP1 (residues 157–167) was obtained from CASLO Laboratory ApS (Denmark). The lyophilized peptide was resuspended directly in NMR buffer and the pH was readjusted to 7.1. The concentration was determined from the volume added to the 10 mg peptide powder. For the phosphorylation reactions carried out in the presence of the peptide, the final concentration of peptide in the sample was 10 µm.

### NMR Spectroscopy and Data Analysis

To monitor the phosphorylation of JIP1 in real‐time, a reference ^1^H‐^15^N HSQC spectrum of JIP1 was initially recorded at 5 °C. Then, 5 µL of active JNK1α1 was added to the NMR tube containing 150 µL of the sample. The phosphorylation kinetics were then monitored by acquiring a series of sensitivity‐enhanced ^1^H‐^15^N HSQC spectra over a 40‐h period at 25 °C (sweep width of 23 ppm in the ^15^N dimension, 320 increments in the ^15^N dimension, 4 scans, recycle delay of 1 s, total acquisition time of 25 min per HSQC). After completing the kinetic measurements, the sample temperature was changed to 5 °C to acquire a ^1^H‐^15^N HSQC spectrum under the same conditions as the reference. This protocol was carried out to measure the phosphorylation kinetics of JIP1^WT^, JIP1^SH3^, JIP1^F‐mut^, JIP1^D‐mut^, JIP1^D‐mut^ + peptide and JIP1^D‐F‐mut^ + peptide. Two biological replicates were carried out for each sample, except for JIP1^SH3^ and JIP1^D‐mut^. All NMR experiments were acquired on 850 or 950 MHz Bruker NMR spectrometers equipped with cryoprobes.

Data processing was performed using NMRPipe^[^ ^87^ ^]^, while spectral analysis was carried out with Sparky^[^ ^88^ ^]^ or in‐house software to extract the noise levels and the intensities of phospho‐peaks across the series of HSQC spectra. The kinetic phosphorylation profiles, *k*(*t*), of the eleven S/T‐P sites in JIP1 were analyzed using:

(2) $$k\left(t\right)=p-p\cdot \exp\left(-rt\right)$$

which accounts for the possibility that phosphorylation may not reach 100% due to limitations in available ATP as JNK1 exhibits a high intrinsic hydrolysis rate. The phosphorylation percentage of each phosphosite was quantified at the end of the time series by measuring the ratio of the peak intensities corresponding to non‐phosphorylated residues in the two ^1^H‐^15^N HSQC spectra recorded before (*I*_0_) and after (*I*) the phosphorylation series. The spectra for quantification of the phosphorylation percentages were acquired at 5 °C to minimize the effects of amide proton exchange on the NMR signal intensities. For S15, T103, S197, T284, S341, and S421, the peaks of the unmodified serine or threonine residues were monitored directly, while for S20, S29, T205, S235 and T448, the peaks of their preceding residues were analyzed to avoid spectral overlap. The phosphorylation rates, λ, for each phosphosite were quantified as the initial slopes derived from the kinetic phosphorylation profiles.

Resonances of phosphorylated residues were assigned using triple resonance experiments of phosphorylated sub‐constructs of JIP1 (JIP1_245‐450_ and JIP1_1‐372_). HNCACB spectra were recorded at 5 °C and at a ^1^H frequency of 950 MHz. The spectrum of JIP1_1‐372_ contained both intra‐ and inter‐residue correlations for Cα and Cβ nuclei, whereas the spectrum of JIP1_245‐450_ contained only the Cβ correlations. The triple resonance experiments enabled unambiguous assignment of the resonances corresponding to phosphorylated residues (Table S1, Supporting Information), which was further confirmed by site‐directed mutagenesis and overlays of ^1^H‐^15^N HSQC spectra of phosphorylated JIP1 sub‐constructs.

### Calculation of Local Effective Concentrations

Conformational ensembles of the JIP1‐JNK1 complex were generated using methods implemented in the MoMA software suite (https://moma.laas.fr).^[^ ^89^ ^]^ For modeling the JNK1‐JIP1 complex, a static structure of JNK1 with the D‐motif bound (PDB 9FT9) with the remainder of the 450‐amino acid long IDR sampling statistical coil conformations avoiding steric clashes with JNK1 and the already built part of the JIP1 chain was considered. The local effective concentration, *C*_eff_, of each residue in JIP1 at the active site of JNK1 was calculated from a structural ensemble containing 400,000 conformations. The values of *C*_eff_ (in m) were calculated according to^[^ ^90^, ^91^ ^]^:

(3) $$C_{\mathrm{eff}}=\frac{3p\left(r\right)}{4\pi r^{3}}\cdot \frac{10^{27}}{N_{A}}$$

where *N*_A_ is Avogadro's constant and *p*(*r*) represents the probability of finding a given residue of JIP1 at the active site of JNK1. This probability was calculated as the ratio of conformations meeting the distance cutoff *r* (in Å). Specifically, for each residue in each conformation of JIP1, the distance between its C_α_ atom and the C_β_ atom of T188 in the “P+1 loop” of JNK1 was calculated. A given residue of JIP1 was considered to be located at the active site if this distance was shorter than 15 Å. For the structural ensembles in which JIP1 was tethered to JNK1 at both the D‐ and F‐motifs, we employed a similar approach, using an extended version of the stochastic sampling method that incorporates loop closure constraints to model the linker region between the motifs.^[^ ^92^ ^]^

## Conflict of Interest

The authors declare no conflict of interest.

## Supporting information



Supporting Information

## Data Availability

The data that support the findings of this study are available from the corresponding author upon reasonable request.
