# Supplementary material for: Sequence‐ and Docking‐Site‐Dependent Contributions to Multi‐Site Phosphorylation of an Intrinsically Disordered MAPK Substrate
Source: Adv Sci (Weinh). 2025 Jun 29;12(35):e03987. doi: 10.1002/advs.202503987 (PMC12463012; doi:10.1002/advs.202503987)
Supplement: Supplementary file 1 — Supporting Information [file ADVS-12-e03987-s001.pdf]

ADVANCED SCIENCE
Open Access

Supporting Information

for *Adv. Sci.*, DOI 10.1002/advs.202503987

Sequence- and Docking-Site-Dependent Contributions to Multi-Site Phosphorylation of an Intrinsically Disordered MAPK Substrate

*Thibault Orand, Elise Delaforge, Marion Chenal, Maud Tengo, Torsten Herrmann, Juan Cortés, Pau Bernadó and Malene Ringkjøbing Jensen**

Supporting Information

Sequence- and Docking-Site-Dependent Contributions to Multi-Site Phosphorylation of an Intrinsically Disordered MAPK Substrate

Thibault Orand[1], Elise Delaforge[1], Marion Chenal[1], Maud Tengo[1],
Torsten Herrmann[1], Juan Cortés[2], Pau Bernadó[3], Malene Ringkjøbing Jensen[1]*

[1]Univ. Grenoble Alpes, CEA, CNRS, IBS, Grenoble, France

[2]LAAS-CNRS, Université de Toulouse, CNRS, 31400, Toulouse, France

[3]Centre de Biologie Structurale (CBS), Université de Montpellier, INSERM, CNRS, Montpellier, France

* To whom correspondence should be addressed

Dr. Malene Ringkjøbing Jensen

E-mail: malene.jensen@ibs.fr

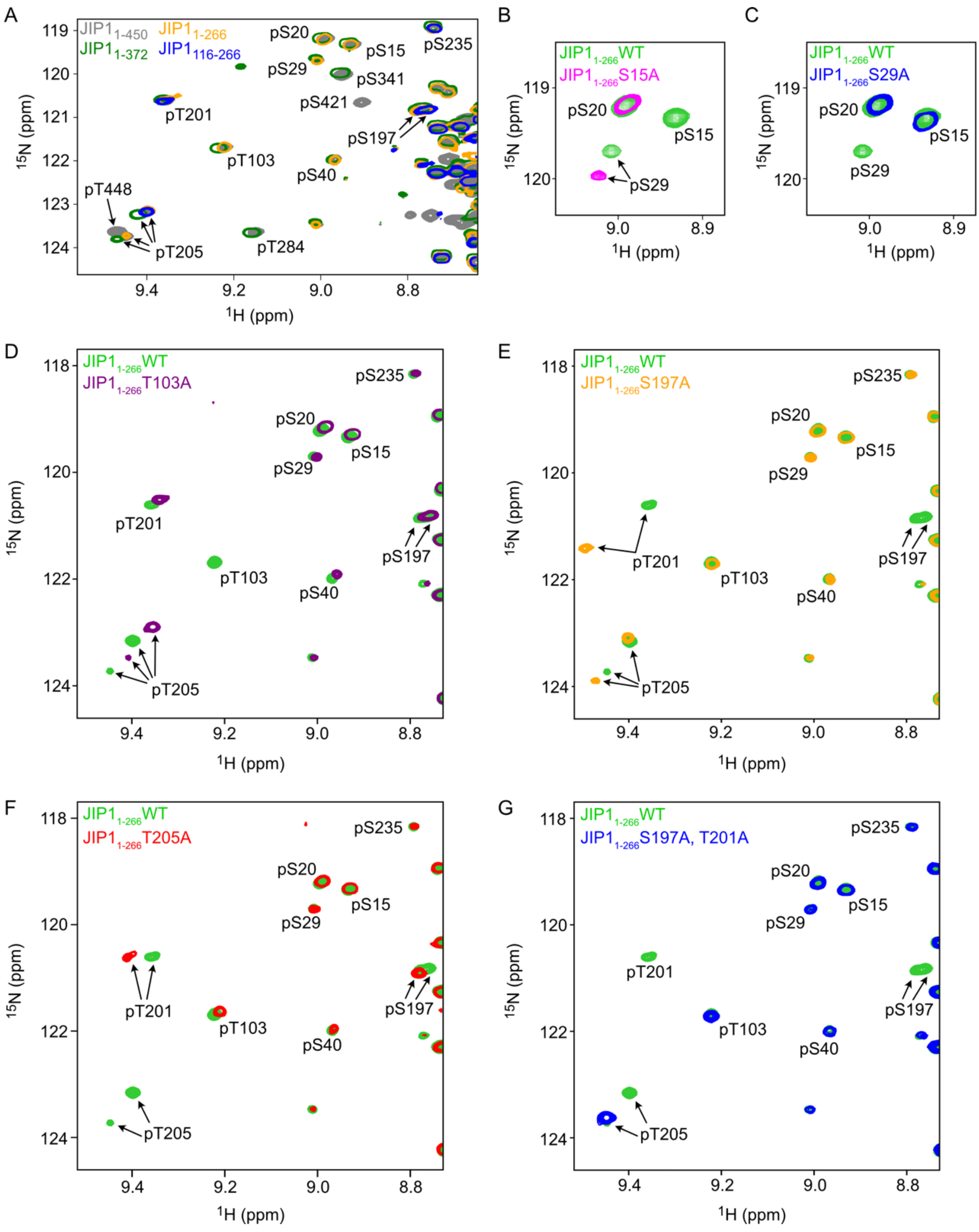

Figure S1. Phosphorylation of different JIP1 constructs by JNK1 and spectral assignment of phosphorylated resonances in JIP1$_{1\text{-}266}$ via mutagenesis. (A) Overlay of the ^{1}H-^{15}N HSQC spectra of phosphorylated JIP1$_{1\text{-}450}$ (grey), JIP1$_{1\text{-}266}$ (orange), JIP1$_{116\text{-}266}$ (blue) and JIP1$_{1\text{-}372}$ (green). Peaks corresponding to phosphorylated residues are labeled. (B-G) Overlay of the ^{1}H-^{15}N HSQC spectra highlighting phosphorylated residues of wild-type JIP1$_{1\text{-}266}$ and its alanine mutants: S15A (B), S29A (C), T103A (D), S197A (E), T205A (F) and S197A/T201A (G). All spectra were recorded at 5°C with a JIP1 concentration of 100 μM.

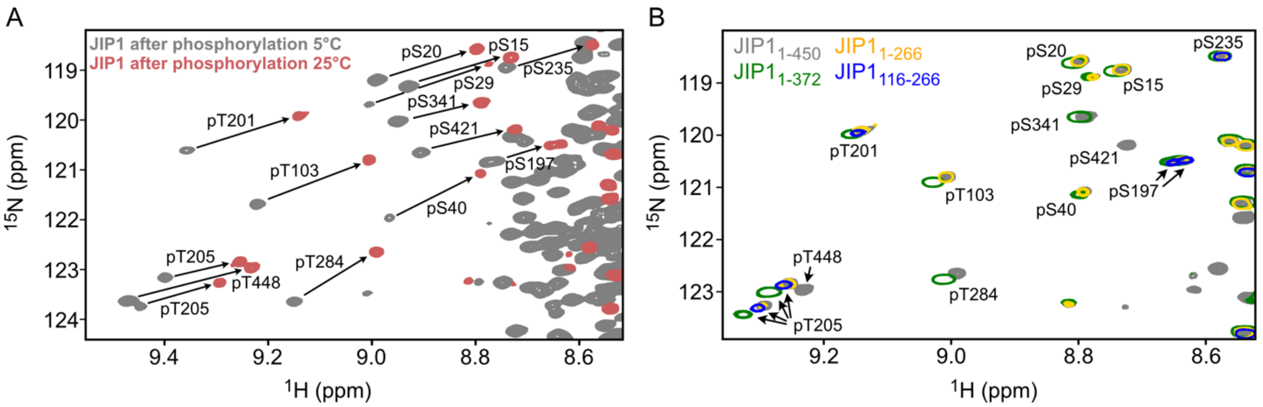

Figure S2. Spectral assignment of resonances corresponding to phosphorylated residues at 25°C. (A) Overlay of the ^{1}H-^{15}N HSQC spectra acquired of phosphorylated JIP1$_{1-450}$ at 5°C (gray) and 25°C (red). (B) Overlay of the ^{1}H-^{15}N HSQC spectra of phosphorylated JIP1$_{1-450}$ (grey), JIP1$_{1-266}$ (orange), JIP1$_{116-266}$ (blue) and JIP1$_{1-372}$ (green). The spectra were acquired at 25°C.

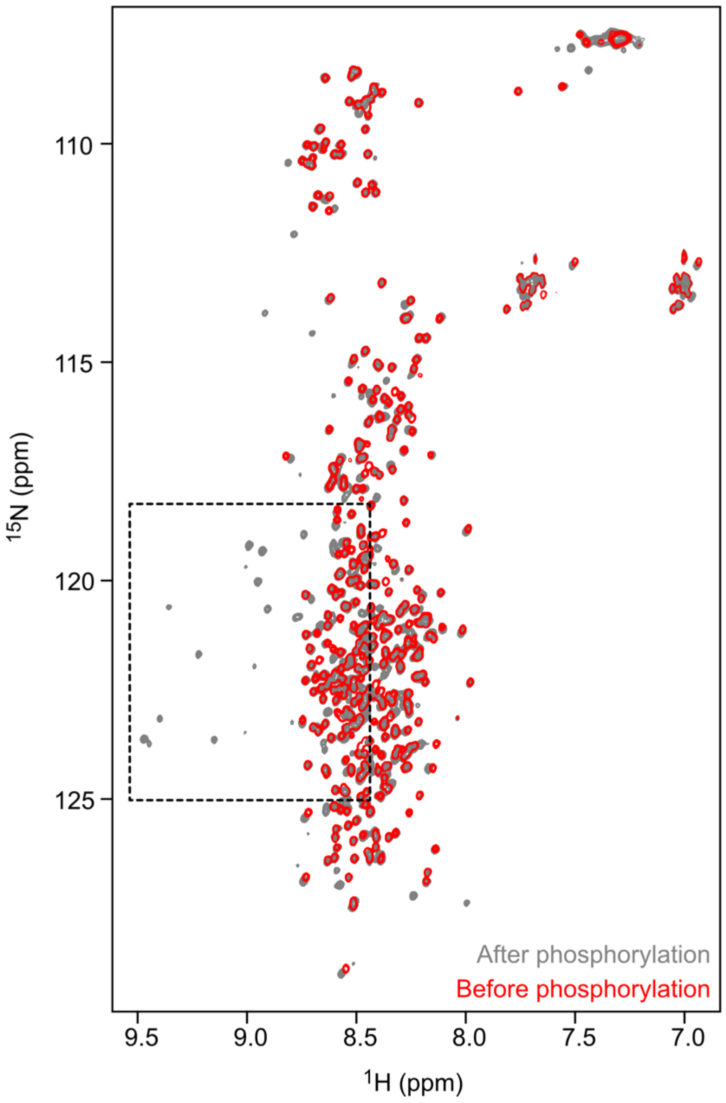

Figure S3. JIP1 remains disordered following multi-site phosphorylation by JNK1. ^{1}H-^{15}N HSQC spectrum of wild-type JIP1$_{1\text{-}450}$ before (red) and after (grey) phosphorylation, showing that the narrow ^{1}H dispersion of the NMR resonances is maintained after phosphorylation, except for resonances corresponding to phosphorylated residues. The dashed box highlights the spectral region, displayed in Figure 1C, which contains the resonances of the phosphorylated residues. The spectra were recorded at 5°C with a JIP1 concentration of 100 µM.

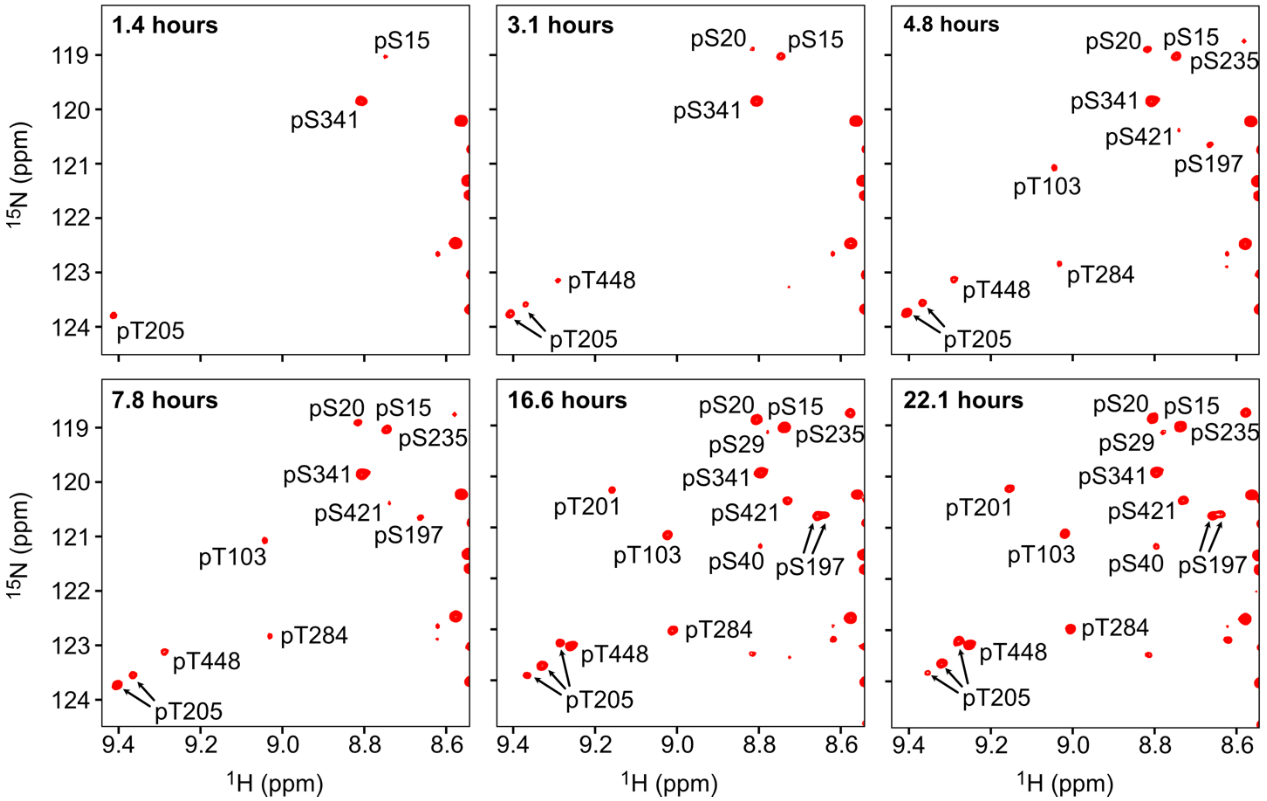

Figure S4. The serine/threonine residues in JIP1 show differential phosphorylation kinetics. ^{1}H-^{15}N HSQC spectra (zoom on the region containing the resonances of phosphorylated residues) acquired at various time points during the phosphorylation kinetics. Active JNK1 was added at time zero hours. The sites, S197 and T205, show two and three distinct resonances, respectively, over the time course. All spectra were recorded at 25°C with a JIP1 concentration of 100 μM.

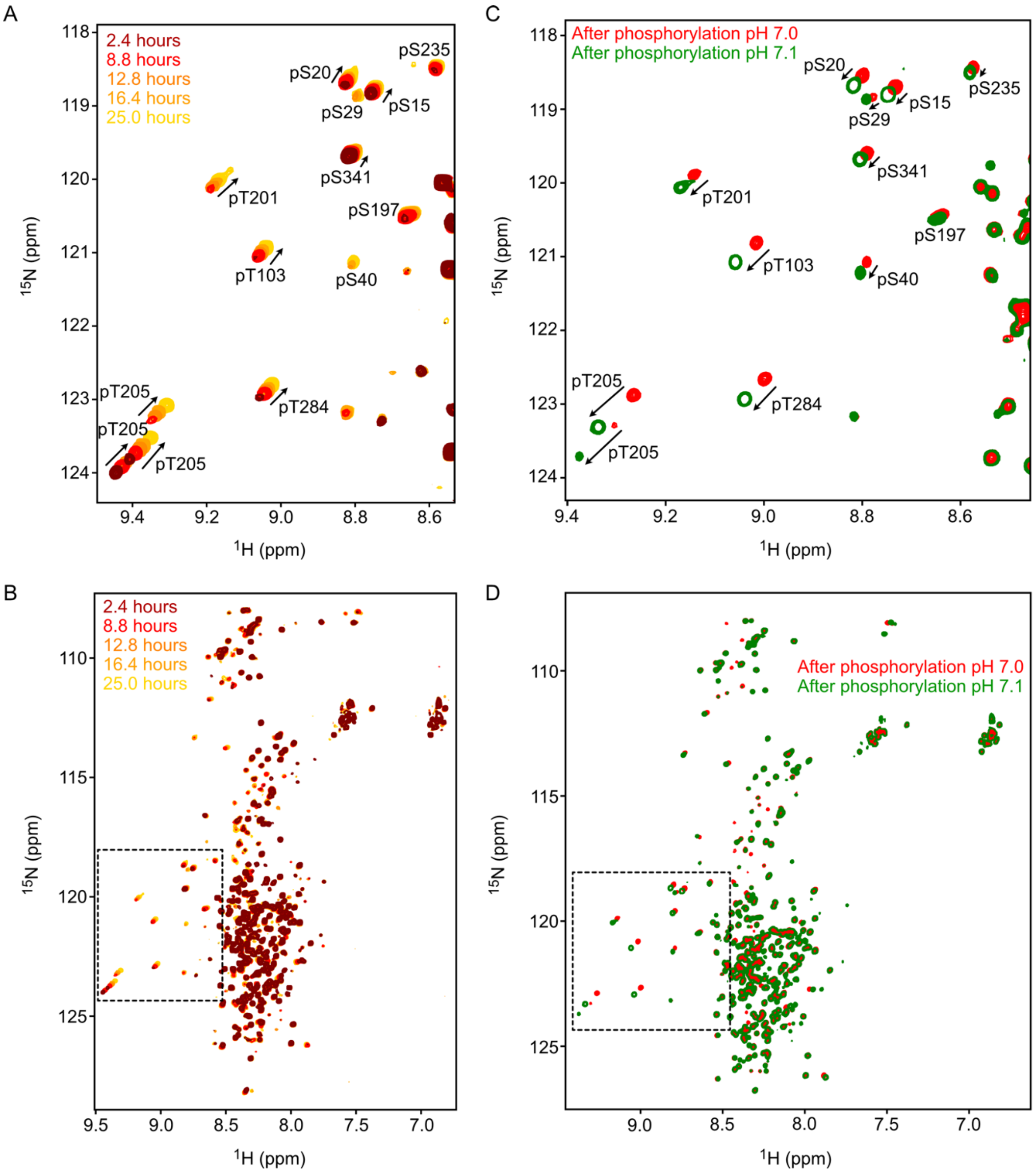

Figure S5. Resonances corresponding to phosphorylated residues undergo chemical shift perturbations during the course of the time series due to a minor decrease in pH. (A, B) Overlay of the ^{1}H-^{15}N HSQC spectra acquired at various time points during the JNK1 phosphorylation reaction of JIP1$_{1\text{-}372}$ at 25°C, showing chemical shift perturbations of phosphorylated residues. Panel A shows a zoom on the resonances corresponding to phosphorylated residues, while panel B displays the full spectra. (C, D) ^{1}H-^{15}N HSQC spectra recorded of JIP1$_{1\text{-}372}$ at 25°C at the end of the phosphorylation reaction (pH 7.0, red), and after readjusting the pH to its initial value before phosphorylation (pH 7.1, green). Panel C shows a zoom on the resonances corresponding to phosphorylated residues, while panel D displays the full spectra.

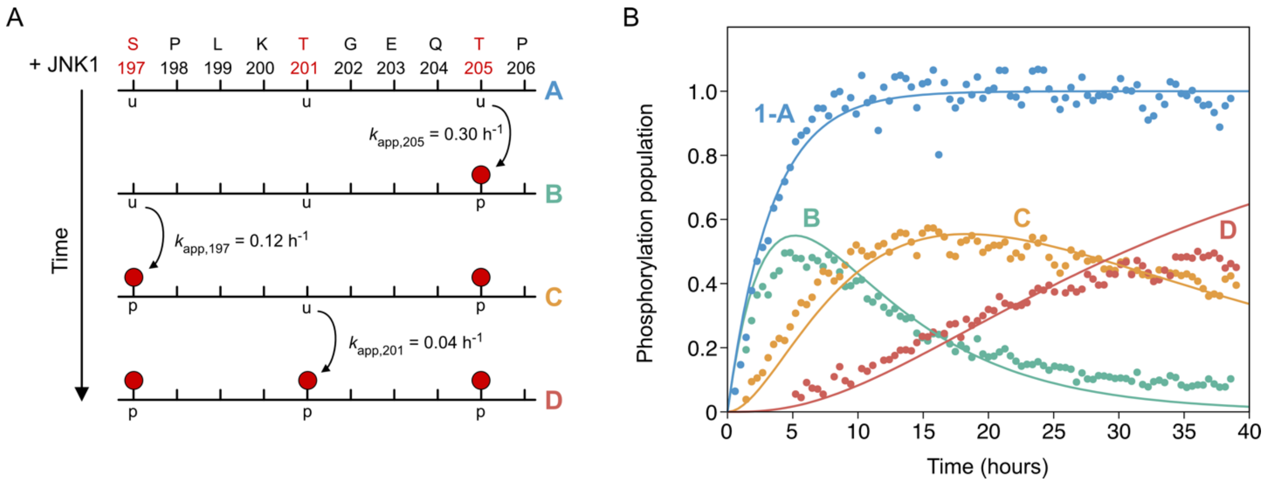

Figure S6. Analysis of apparent phosphorylation rates in the phosphorylation cluster composed of S197, T201 and T205. (A) Sequence of JIP1 encompassing the S197-T201-T205 phosphorylation cluster. The order of phosphorylation is shown, and the apparent phosphorylation rate constants are indicated as obtained from an analysis of the time dependence of the resonance intensities of T205 at 25°C. (B) Intensities of T205 resonances during the 40-hour time series at 25°C. Three resonances appear during the course of phosphorylation corresponding to a single-phosphorylated state of JIP1 (at T205, green circles), a double-phosphorylated state (at T205 and S197, orange circles) and a triple-phosphorylated state (at T205, S197 and T201, red circles). The blue circles correspond to the sum of the intensities of the three resonances of T205. Lines correspond to an analysis of the time-dependence of the percentage of phosphorylation according to a linear sequence of irreversible reactions: $A \xrightarrow{k_{\text{app},205}} B \xrightarrow{k_{\text{app},197}} C \xrightarrow{k_{\text{app},201}} D$. Here, A corresponds to the unphosphorylated form of JIP1, B to the single-phosphorylated form, C to the double-phosphorylated form, and finally D to the triple-phosphorylated form. The apparent phosphorylation rate constants were determined from the following equations describing the time-dependence of the percentage of phosphorylation:

$$A(t) = e^{-k_{\text{app},205}t} \qquad \text{eq S.1}$$

$$B(t) = \frac{k_{\text{app},205}}{k_{\text{app},197} - k_{\text{app},205}}\left(e^{-k_{\text{app},205}t} - e^{-k_{\text{app},197}t}\right) \qquad \text{eq S.2}$$

$$C(t) = \frac{k_{\text{app},205}k_{\text{app},197}}{(k_{\text{app},197} - k_{\text{app},205})(k_{\text{app},201} - k_{\text{app},205})}\left(e^{-k_{\text{app},205}t} - e^{-k_{\text{app},201}t}\right) - \frac{k_{\text{app},205}k_{\text{app},197}}{(k_{\text{app},201} - k_{\text{app},197})(k_{\text{app},197} - k_{\text{app},205})}\left(e^{-k_{\text{app},197}t} - e^{-k_{\text{app},201}t}\right) \qquad \text{eq S.3}$$

$$D(t) = 1 - A(t) - B(t) - C(t) \qquad \text{eq S.4}$$

The rate constant, $k_{\text{app},205}$, was initially determined from equation S.1. This value was then fixed to determine $k_{\text{app},197}$ from equation S.2. Subsequently, both of these rate constants were fixed to determine $k_{\text{app},201}$ from equation S.3. Finally, equation S.4 was employed to back-calculate the expected percentage of phosphorylation for the triple-phosphorylated state.

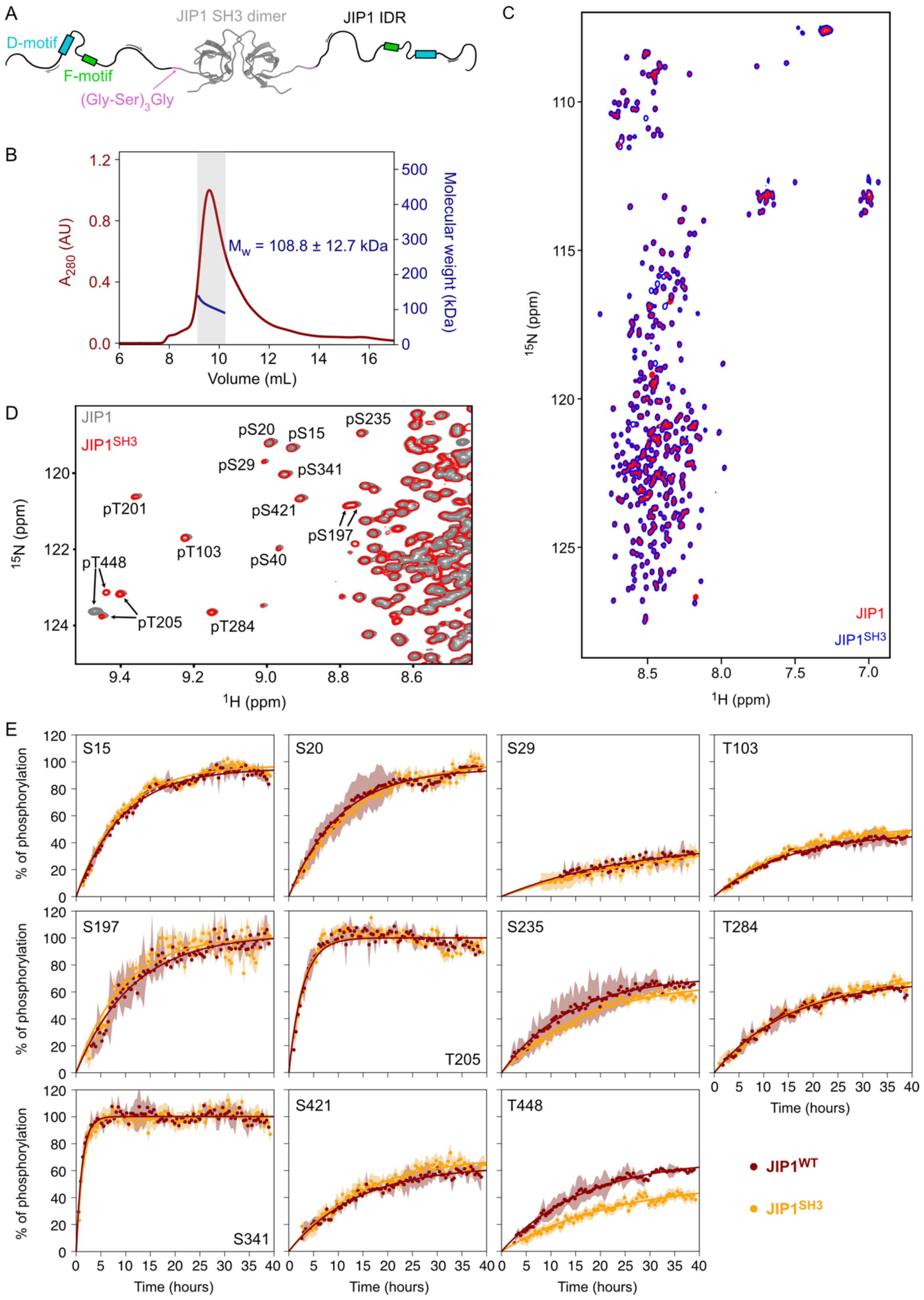

Figure S7. Dimerization does not impact the site-specific phosphorylation rates in the IDR of JIP1. (A) Schematic representation of the engineered dimeric construct of JIP1 (JIP1^{SH3}). (B)

Determination of the molecular weight of JIP1^{SH3} from size exclusion chromatography (SEC) coupled with multiangle laser light scattering (MALLS). The SEC elution profile (UV absorption at 280 nm, red curve, left axis) and molecular mass calculated from light scattering and refractometry data (blue, right axis) confirm that JIP1^{SH3} forms a dimer in solution (expected molecular weight for a dimer: 111 kDa). The region used for molecular weight calculation is highlighted in gray. (C) Overlay of the ^{1}H-^{15}N HSQC spectra of JIP1 (monomer, red) and JIP1^{SH3} (dimer, blue) recorded at 5°C with a JIP1 concentration of 100 μM of JIP1 (monomer) and 50 μM of JIP1^{SH3} (dimer). The spectral similarity suggests that dimerization via the SH3 domain does not alter the conformational ensemble of the JIP1 IDR. (D) Overlay of the ^{1}H-^{15}N HSQC spectra recorded at 5°C of JIP1^{WT} (gray) and JIP1^{SH3} (red) after JNK1 phosphorylation. The spectra show that dimerization does not alter the overall phosphorylation landscape. (E) Kinetic phosphorylation profiles for each of the eleven S/T-P sites in JIP1 derived from NMR resonance intensities normalized to the percentage of phosphorylation at the end of the time series. Data are shown for JIP1^{WT} (red, average of two biological replicates, standard deviation in light red) and for JIP1^{SH3} (orange, single experiment, uncertainty estimated as twice the noise level in each plane, light orange shading). The intensities were analyzed using Eq. 2 (solid lines). The results show that dimerization does not influence the phosphorylation kinetics, except for T448, which is located near the dimerization domain. The ^{1}H-^{15}N HSQC spectra were recorded at 25°C and at a concentration of 100 μM for JIP1^{WT} and 50 μM for JIP1^{SH3}.

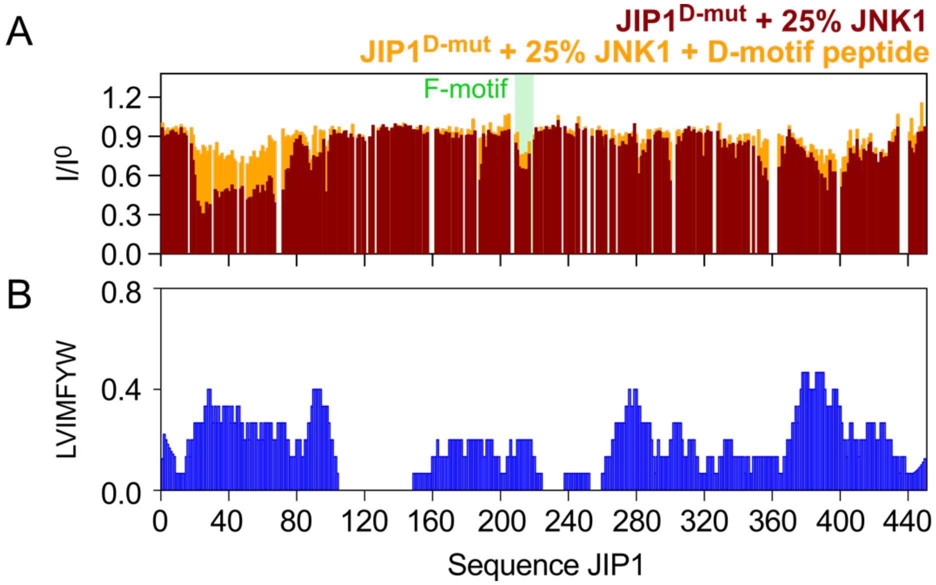

Figure S8. Transient interaction of hydrophobic clusters in JIP1$^{D\text{-}mut}$ with the D-motif recruitment site (DRS) of JNK1. (A) NMR signal intensity ratios (I/I^0) of JIP1$^{D\text{-}mut}$ measured in the absence (I^0) and presence (I) of inactive JNK1 at a 25% molar ratio. Data are shown for two different conditions: without (red) and with (yellow) the D-motif peptide of JIP1. The addition of the D-motif peptide displaces JNK1 from JIP1$^{D\text{-}mut}$, demonstrating that the unoccupied DRS in JIP1$^{D\text{-}mut}$ recognizes certain hydrophobic clusters within JIP1. The concentration of JIP1$^{D\text{-}mut}$ and the D-motif peptide was 50 and 250 µM, respectively. (B) Distribution of hydrophobic residues (Leu, Val, Ile, Met, Phe, Tyr and Trp) along the JIP1 sequence. Hydrophobic residues were assigned a value of +1, with the distribution smoothed over a 15-residue window (blue).

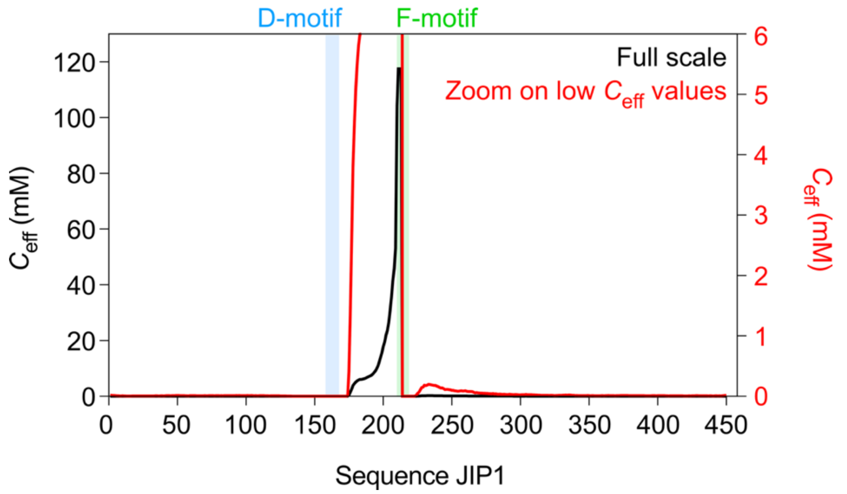

Figure S9. Local effective concentrations, C_{eff}, for all residues in JIP1 calculated from structural ensembles of the JIP1-JNK1 complex with JIP1 anchored to JNK1 via both the D- and F-motifs. The resulting C_{eff} values are presented using two different y-axis scales for clarity.

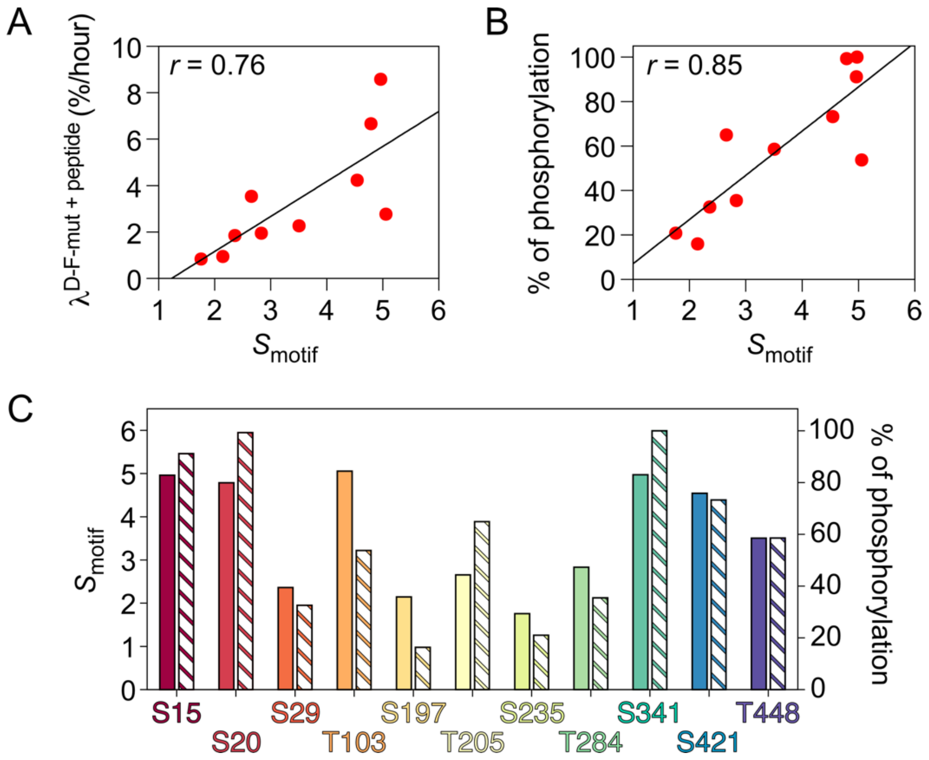

Figure S10. Correlation between motif scores and site-specific phosphorylation rates and percentages. (A) Scatter plot showing the correlation between calculated motif scores, S_{motif}, and site-specific phosphorylation rates of JIP1$^{\mathrm{D\text{-}F\text{-}mut}}$ + peptide (λ, defined as the initial slope of the kinetic phosphorylation profiles). The plot shows the same data as in Fig. 5B except that the data for S341 were excluded. (B) Scatter plot showing the correlation between motif scores and the percentage of phosphorylation at infinite time of JIP1$^{\mathrm{D\text{-}F\text{-}mut}}$ + peptide. For both panel A and B, the Pearson correlation coefficient is given. (C) The same data as in panel B, but plotted as a bar plot. Motif scores, S_{motif}, calculated for the eleven S/T-P sites in JIP1 (left y-axis, filled bars) are compared to the percentage of phosphorylation of JIP1$^{\mathrm{D\text{-}F\text{-}mut}}$ + peptide (right y-axis, hatched bars).

Table S1. Chemical shift assignments (pH 7.0 and 5°C) of phosphorylated residues in JIP1. S197 and T205 show two and three distinct resonances, respectively, over the course of the time series.

	JIP1			
	**N (ppm)**	**H^N (ppm)**	**Cα (ppm)**	**Cβ (ppm)**
**pS15**	119.33	8.93	56.09	64.62
**pS20**	119.20	8.99	56.30	64.67
**pS29**	119.69	9.01	56.15	64.57
**pS40**	121.96	8.97	58.02	65.64
**pT103**	121.69	9.22	61.22	72.33
**pS197#1**	120.86	8.78	56.40	64.52
**pS197#2**	120.82	8.76	56.40	64.52
**pT201**	120.61	9.36	63.65	72.00
**pT205#1**	123.96	9.48	61.14	72.70
**pT205#2**	123.74	9.45	61.06	72.65
**pT205#3**	123.16	9.40	61.03	72.70
**pS235**	118.95	8.74	56.63	64.81
**pT284**	123.65	9.15	60.83	72.59
**pS341**	123.04	8.95	56.78	64.28
**pS421**	120.65	8.91	-	64.60
**pT448**	123.64	9.47	-	72.81

Table S2. Phosphorylation percentages and rates for the 11 phosphosites in JIP1. Percentages were measured 40 hours after addition of the JNK1 kinase. Phosphorylation rates were calculated as the initial slopes of the averaged (over two independent replicates) kinetic phosphorylation profiles. Error bars represent standard deviations from the duplicate experiments. A † symbol indicates that both replicates showed 100% phosphorylation. Data are reported for three experimental conditions: $JIP1^{WT}$, $JIP1^{F\text{-}mut}$ and $JIP1^{D\text{-}F\text{-}mut}$ + peptide.

	% phosphorylation	λ (%/hour)
	**$JIP1^{WT}$**	
**S15**	94.2 ± 1.5	10.15 ± 0.23
**S20**	94.1 ± 1.8	9.25 ± 3.90
**S29**	29.0 ± 0.1	1.53 ± 0.19
**T103**	47.8 ± 3.1	3.23 ± 0.17
**S197**	100.0†	9.00 ± 3.16
**T205**	100.0†	35.38 ± 9.03
**S235**	71.4 ± 2.3	5.18 ± 2.83
**T284**	63.5 ± 2.9	4.54 ± 1.19
**S341**	100.0†	83.25 ± 17.01
**S421**	59.9 ± 7.2	4.60 ± 0.63
**T448**	60.8 ± 1.1	4.15 ± 1.31
	**$JIP1^{F\text{-}mut}$**	
**S15**	92.0 ± 2.4	9.35 ± 1.02
**S20**	89.9 ± 4.4	9.35 ± 1.37
**S29**	28.2 ± 2.3	1.48 ± 0.06
**T103**	38.5 ± 5.8	2.83 ± 0.79
**S197**	97.1 ± 4.0	6.61 ± 0.63
**T205**	100.0†	17.69 ± 2.90
**S235**	57.3 ± 0.3	3.88 ± 0.63
**T284**	56.0 ± 2.9	3.58 ± 0.28
**S341**	100.0†	87.17 ± 3.66
**S421**	62.7 ± 0.8	4.58 ± 1.07
**T448**	48.8 ± 2.2	2.92 ± 0.47
	**$JIP1^{D\text{-}F\text{-}mut}$ + peptide**	
**S15**	89.1 ± 1.5	8.58 ± 0.79
**S20**	92.5 ± 6.2	6.66 ± 0.14
**S29**	29.2 ± 1.7	1.85 ± 0.33
**T103**	46.9 ± 0.6	2.77 ± 0.01
**S197**	14.6 ± 0.4	0.95 ± 0.09
**T205**	57.6 ± 0.1	3.54 ± 0.01
**S235**	16.6 ± 1.2	0.84 ± 0.10
**T284**	31.5 ± 3.3	1.95 ± 0.28
**S341**	100.0†	84.60 ± 2.02

**S421**	66.0 ± 0.5	4.23 ± 0.31
**T448**	46.1 ± 0.8	2.27 ± 0.01
